# Calcifying fibrous tumor of the ileum resected by single-port laparoscopic surgery: a case report

**DOI:** 10.1186/s40792-022-01423-8

**Published:** 2022-04-13

**Authors:** Kazuya Takabatake, Tomohiro Arita, Yoshiaki Kuriu, Hiroki Shimizu, Jun Kiuchi, Wataru Takaki, Hirotaka Konishi, Yusuke Yamamoto, Ryo Morimura, Atsushi Shiozaki, Hisashi Ikoma, Takeshi Kubota, Hitoshi Fujiwara, Kazuma Okamoto, Yuta Sonobe, Noriyuki Tanaka, Eiichi Konishi, Eigo Otsuji

**Affiliations:** 1 Division of Digestive Surgery, Department of Surgery, Kyoto Prefectural University of Medicine, 465 Kajii-cho, Kamigyo-ku, Kyoto, 6028566 Japan; 2 Department of Surgical Pathology, Kyoto Prefectural University of Medicine, 465 Kajii-cho, Kamigyo-ku, Kyoto, 6028566 Japan

**Keywords:** Calcifying fibrous tumor, Ileal neoplasm, Single-port laparoscopic surgery

## Abstract

**Background:**

Calcifying fibrous tumors (CFTs) are rare benign tumors. Because CFTs sometimes relapse, radical resection with adequate margins is necessary. We report a case of ileal CFT resected using single-port laparoscopic surgery.

**Case presentation:**

A 33-year-old man presented with chief complaints of abdominal pain and vomiting. Computed tomography demonstrated a 45-mm-sized pelvic mass with partial calcification in the ileum. The patient was diagnosed with an ileal tumor, and partial resection of the ileum was performed using the single-port laparoscopic technique. Pathologic findings revealed hypocellular spindle cells with dense hyalinized collagen, interspersed calcification, and infiltration of lymphoplasmacytic cells. Immunohistochemical analysis showed that the factor XIIIa was positive and other tumor-specific markers were negative. Based on these findings, the tumor was finally diagnosed as a CFT.

**Conclusions:**

Although CFT is benign, multifocal and recurrent CFTs have been reported. Therefore, careful intraperitoneal observation and curative resection are necessary. Single-port laparoscopic surgery is acceptable, both in terms of curability and minimal invasiveness.

## Background

Calcifying fibrous tumors (CFTs) are rare benign tumors characterized by hypocellular spindle cells, hyalinized collagen, lymphoplasmacytic infiltrate, and scattered calcification, and are categorized as bone and soft-tissue tumors [1]. However, CFTs can occur in any part of the body, including the gastrointestinal tract [2].

A curative resection of CFTs with a sufficient margin is necessary, and in most cases, laparotomy has been performed [2]. In recent years, however, there have been advances in laparoscopic surgery, and laparoscopic resection of CFTs has also been reported in some cases [3–5]. Single-port laparoscopic surgery, in which all laparoscopic working ports approach the abdominal wall through the same incision, has been developed as a much less invasive method and has been reported to provide better cosmetic results, reduced postoperative pain, and improved oncological safety [6].

Herein, we present a case of ileal CFT resected using single-port laparoscopic surgery.

## Case presentation

**Fig. 1:**
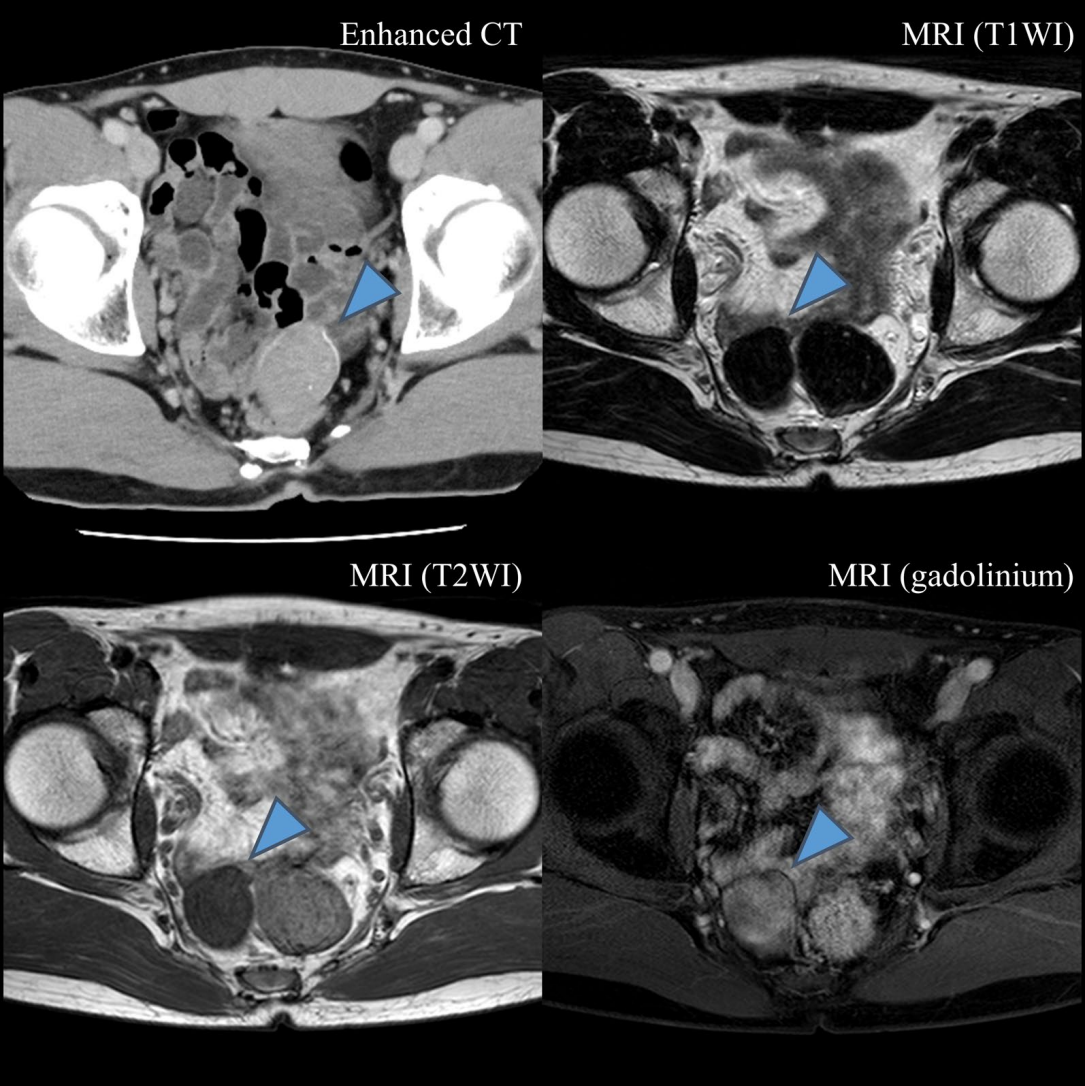
CT and MRI images. A 45-mm-sized mass (blue arrow head) with partial calcification was observed in the ileum on enhanced CT. The mass was hypointense signal on both T1WI and T2WI, and isointense signal on gadolinium-enhanced T1WI. *CT* computed tomography, *MRI* magnetic response image, *T1WI* T1-weighted image, *T2WI* T2-weighted image

A 33-year-old man without a previous medical or surgical history presented with chief complaints of abdominal pain and vomiting. Physical examination revealed tenderness in the lower abdomen. Laboratory data were unremarkable: C-reactive protein level, 0.16 mg/dL; white blood cell count, 9600 /μL; neutrophil count, 91.3%; and lymphocyte count, 5.4%. Ultrasonography and radiography revealed no findings that could cause abdominal pain. Contrast-enhanced computed tomography (CT) demonstrated a 45-mm-sized mass with partial calcification in the ileum (Fig. 1), without any signs of invagination, obstruction, or volvulus. No other findings suggesting the cause of abdominal pain were observed on CT. The patient was diagnosed with an ileal tumor. After the examination, the patient’s condition improved spontaneously. Several weeks later, for detailed examination of the tumor, magnetic resonance imaging (MRI) was performed, which revealed a tumor with hypointense signal on both T1-weighted (T1WI) and T2-weighted images (T2WI), and isointense signal on gadolinium-enhanced T1WI (Fig. 1). Based on these findings, the differential diagnoses were a gastrointestinal stromal tumor, chronic distending hematoma, leiomyoma, and CFT.

**Fig. 2:**
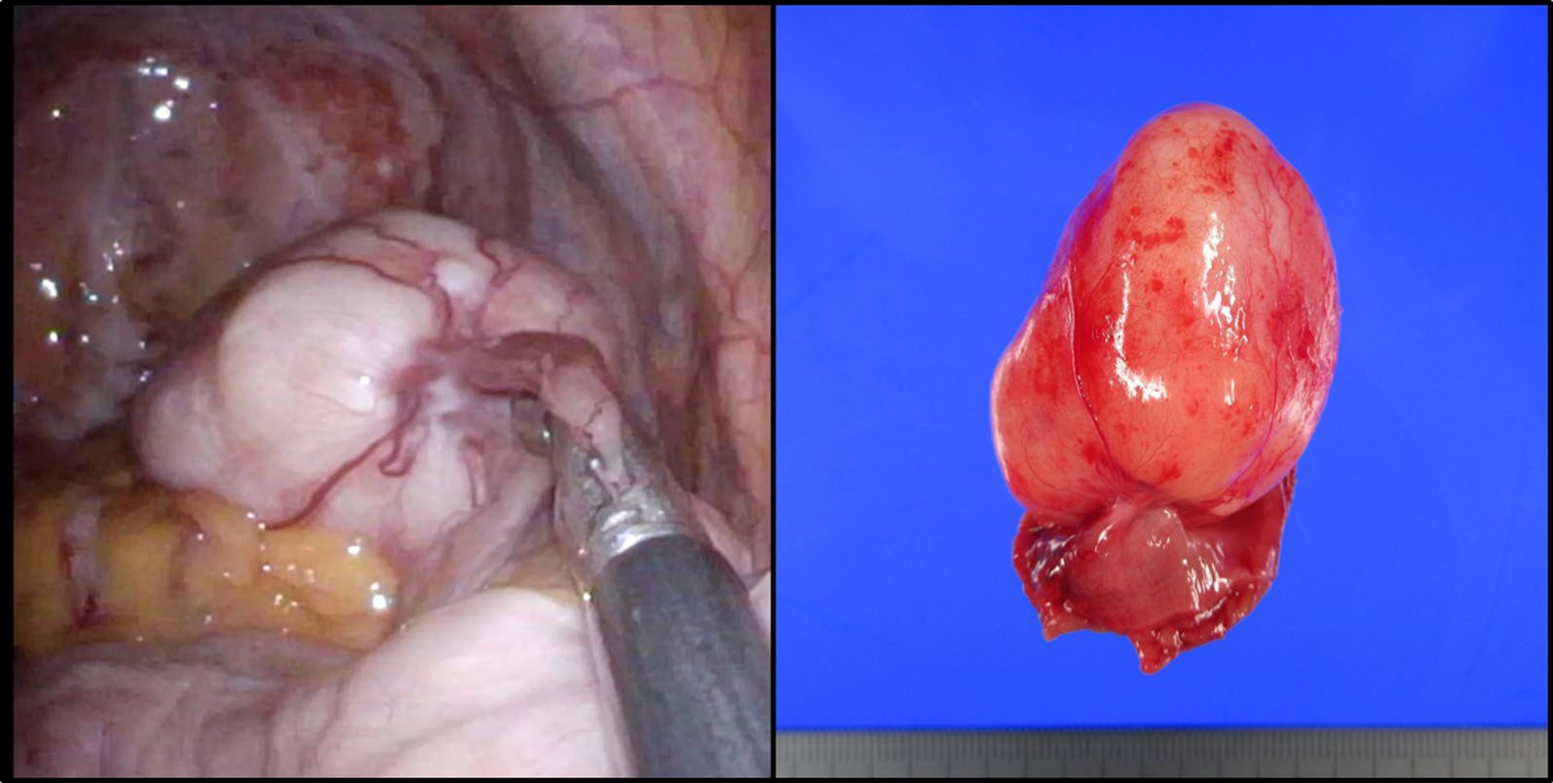
Intraoperative findings. A white-colored tumor was found in the ileum. The tumor was pedunculated and located on the antimesenteric side

**Fig. 3:**
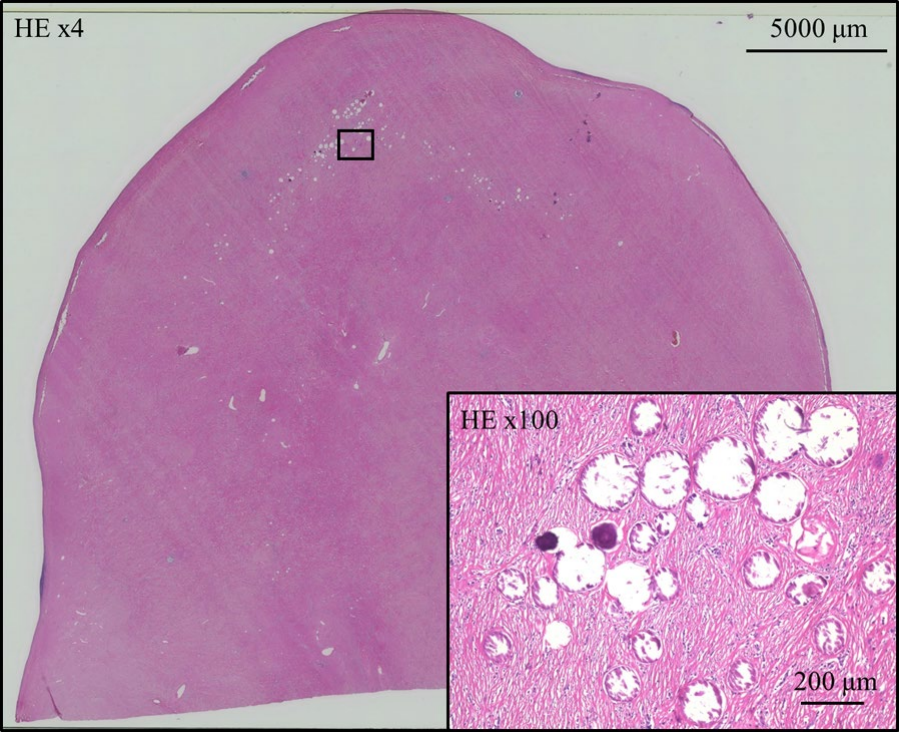
Microscopic findings. A tumor was well-circumscribed and unencapsulated in a low-power field. A few spindle cells and lymphoplasmacytic infiltration were observed with dense hyalinized collagen and interspersed calcification in the background in a high-power field. *HE* hematoxylin–eosin

Laparoscopic surgery was performed for the pathological diagnosis and treatment. Two 5-mm ports were placed through a vertical 4-cm skin incision in the umbilicus using EZ access and Lap Protector (Hakko Medical, Nagano, Japan). Intraoperatively, a white-colored tumor was found in the ileum, 100 cm from the terminal ileum (Fig. 2). Careful observation of the entire abdominal cavity did not detect any other tumors, lymph node metastasis, or dissemination. After careful observation, the tumor was lead extracorporeally through the umbilical incision and a partial resection of the ileum was performed. Reconstruction was performed with a functional end-to-end anastomosis. Macroscopic findings revealed that the tumor was pedunculated and located on the antimesenteric side. Microscopic findings revealed that the tumor extended from the muscularis propria to the subserosa. A few spindle cells and infiltration of lymphoplasmacytic cells were observed with dense hyalinized collagen and interspersed calcification in the background (Fig. 3). Immunohistochemical findings revealed negative or nearly negative results for CD34, c-kit, DOG-1, desmin, S100, anaplastic lymphoma kinase, vimentin and smooth muscle actin. Factor-XIIIa was positive. The MIB-1 labeling index was less than 1%. Over 40% of the plasma cells in the stroma were IgG4 positive. The tumor was eventually diagnosed as a CFT. The patient was discharged on postoperative day 7 without any complications. No recurrence has been observed in the 6 months since the surgery.

## Discussion

CFT was first described as “childhood fibrous tumor with psammoma bodies” by Rosenthal et al. in 1988 [7]. CFTs can originate from anywhere in the gastrointestinal tract, including the small intestine. Pezhouh et al. reported a total of 13 CFT cases in the small intestine out of 28 cases of CFTs originating from the gastrointestinal tract and most cases were discovered incidentally [8]. However some cases were accompanied by acute abdominal symptoms and a summary of previous reports is shown in Table 1. [3–5, 9–19]. In this case, CT for abdominal symptoms detected the mass. Although CT findings did not show any evidence of obstruction, invagination, or volvulus caused by the mass, these phenomena may have occurred temporarily because other findings causing digestive symptoms were not observed. Temporary volvulus of the tumor with a stalk may be released immediately. Therefore, the possibility of CFTs causing acute abdominal symptoms should be considered.

**Table 1:** Reported cases and our case of small intestine CFTs

Case	Author	Year	Age, Sex	Clinical presentation	Number of lesions	Location in small intestine	Tumor size (cm)	Surgery methods	Recurrence	Follow up
1	Chen	2003	17, female	Abdominal pain	Multiple	Serosa	< 2.0	Laparotomy	No	19 years
2	Chen	2003	17, female	Abdominal pain	Multiple	Serosa	< 2.0	Laparotomy	No	17 years
3	Murakami et al.	2006	58, female	Abdominal pain Vomiting	Single	Extramural	1.8	Laparoscopy	No	Not documented
4	Liang et al.	2007	25, female	Abdominal pain Vomiting	Multiple	Serosa	Not documented	Laparotomy	No	18 months
5	Emanuel et al.	2008	20, male	Invagination	Single	Intramural	2.0	Laparotomy	No	Not documented
6	Emanuel et al.	2008	38, female	Abdominal pain	Single	Subserosa	3.3	Not documented	No	Not documented
7	Emanuel et al.	2008	30, female	Nothing	Single	Subserosa	0.5	Not documented	No	Not documented
8	Emanuel et al.	2008	35, male	Nothing	Single	Subserosa	0.5	Not documented	No	Not documented
9	Giardino et al.	2011	45, male	Abdominal pain	Single	Extramural	5.0	Laparotomy	No	12 months
10	Tseng et al.	2012	30, male	Abdominal pain Vomiting	Multiple	Serosa Mesentery	< 1.0	Laparotomy	No	4 months
11	Takeji et al.	2013	30, female	Invagination	Single	Intramural	2.0	Laparotomy	No	Not documented
12	Valladolid et al.	2014	25, female	Invagination	Single	Intramural	1.9	Laparotomy	No	Not documented
13	Wesecki et al.	2014	27, male	Abdominal pain	Single	Mesentery	6.0	Laparotomy	No	7 years
14	Minami et al.	2015	69, male	Abdominal pain	Single	Intramural	1.0	Laparotomy	No	12 months
15	Luques et al.	2017	24, female	Nothing	Single	Subserosa	4.5	Laparotomy	No	Not documented
16	Sotiriou et al.	2018	54, female	Invagination	Single	Extramural	2.1	Laparotomy	No	14 months
17	Hort et al.	2020	20, male	Abdominal pain	Single	Extramural	6.0	Laparoscopy	No	Not documented
18	Nishina et al.	2020	65, female	Nothing	Single	Serosa	0.5	Laparoscopy	No	9 months
19	Our case		33, male	Abdominal pain Vomiting	Single	Extramural	4.5	Laparoscopy	No	6 months

CFT is a round hyper- or hypodense mass with calcification on CT, hypointense signal on T1WI and T2WI, and isointense signal on gadolinium-enhanced T1WI [2]. Microscopically, hypocellular spindle cells are observed against the background of abundant hyalinized collagen, along with scattered calcifications and lymphoplasmacytic infiltrates [8, 20]. Immunohistochemically, CFT is positive for Factor XIIIa, vimentin, and CD34, and negative for c-kit, DOG-1, desmin, S100, anaplastic lymphoma kinase, and smooth muscle actin [2, 21]. In this case, CT detected a hypointense mass with calcification, and MRI demonstrated that the mass was hypointense on T1WI and T2WI and isointense on gadolinium-enhanced T1WI. These findings are similar to those of previous reports. Microscopic findings of this case, characterized by hypocellular spindle cells, dense hyalinized collagen, interspersed calcifications, and lymphoplasmacytic infiltrates, were also consistent with previous reports. As for the immunohistochemical findings, factor XIIIa was positive and other tumor specific markers were negative. In addition, the MIB-1 index was low, suggesting a low cell growth potential. These results led to the diagnosis of CFT. Although small intestine CFT is rare and similar to other tumors such as GIST, it is important to consider this entity based on the imaging and pathological findings to avoid misdiagnosis.

CFT is a benign tumor and no recurrence of small intestine CFTs was reported (Table 1), while the recurrence rate of all types of CFTs is reported to be 10% [2]. Therefore, complete surgical resection is required. Although no cases of distant metastasis have been reported, some reports have shown multifocal lesions [9, 10, 13]. In addition, as CFTs are often located in the peritoneum or other organs, including the gastrointestinal tract in the abdomen [2], observation of the entire intraperitoneal region is important. Laparoscopic surgery is favorable for extensive intraabdominal observation through a small incision when compared with open laparotomy. Furthermore, we used a single-port laparoscopic technique to observe the entire abdominal cavity and resect the tumor in this case. Single-port laparoscopic surgery is cosmetically superior to multiport surgery [6]. Although single-port surgery requires technical training, the safety of single-port laparoscopic surgery has been reported to be compatible with conventional surgery [6]. Additional port placement can overcome the difficulty of single-port surgery. Therefore, single-port laparoscopic surgery for small intestine tumors, including CFT, is a reasonable method in terms of safety, reliability, and minimal invasiveness.

In summary, CFT is a rare benign lesion that may cause acute abdominal pain. After careful consideration based on the CT, MRI, or microscopic findings, single-port laparoscopic surgery is appropriate in terms of curability and minimal invasiveness.

## Data Availability

Not applicable.

## Abbreviations

CFT: Calcifying fibrous tumor
CT: Computed tomography
MRI: Magnetic resonance imaging
T1WI: T1-weighted image
T2WI: T2-weighted image
